# Supplementary material for: Ultralow Voltage Operation of p‐ and n‐FETs Enabled by Self‐Formed Gate Dielectric and Metal Contacts on 2D Tellurium
Source: Adv Mater. 2025 Apr 8;37(20):2418142. doi: 10.1002/adma.202418142 (PMC12087726; doi:10.1002/adma.202418142)
Supplement: Supplementary file 1 — Supporting Information [file ADMA-37-2418142-s001.docx]

Supplementary Information for:

Ultralow Voltage Operation of p- and n-FETs Enabled by Self-Formed Gate Dielectric and Metal Contacts on 2D Tellurium

Chang Niu^1,2,†^, Linjia Long^1,2,†^, Yizhi Zhang^3^, Zehao Lin^1,2^, Pukun Tan^1,2^, Jian-Yu Lin^1,2^, Wenzhuo Wu^4^, Haiyan Wang^3^ and Peide D. Ye^1,2,^*

^1^*Elmore Family School of Electrical and Computer Engineering, Purdue University, West Lafayette, IN 47907, United States.*

^2^*Birck Nanotechnology Center, Purdue University, West Lafayette, IN 47907, United States.*

^3^*School of Materials Science and Engineering, Purdue University, West Lafayette, Indiana 47907, United States.*

^4^*School of Industrial Engineering, Purdue University, West Lafayette, IN 47907, United States.*

†These authors contributed equally to this work: Chang Niu, Linjia Long

*Correspondence and requests for materials should be addressed to P. D. Y. (yep@purdue.edu)

**List of contents:**

**Supplementary figures:**

**Figure S1. Intimate NiTe_x_-Ni contacts.**

**Figure S2.** **Top-gate and back-gate dependence of the transfer characteristics.**

**Figure S3. Room temperature device behavior with Ti and Al as gate.**

**Figure S4.** **Schottky barrier extraction for n-FET and p-FET.**

**Figure S5.** **Noise margin extraction of the CMOS inverter.**


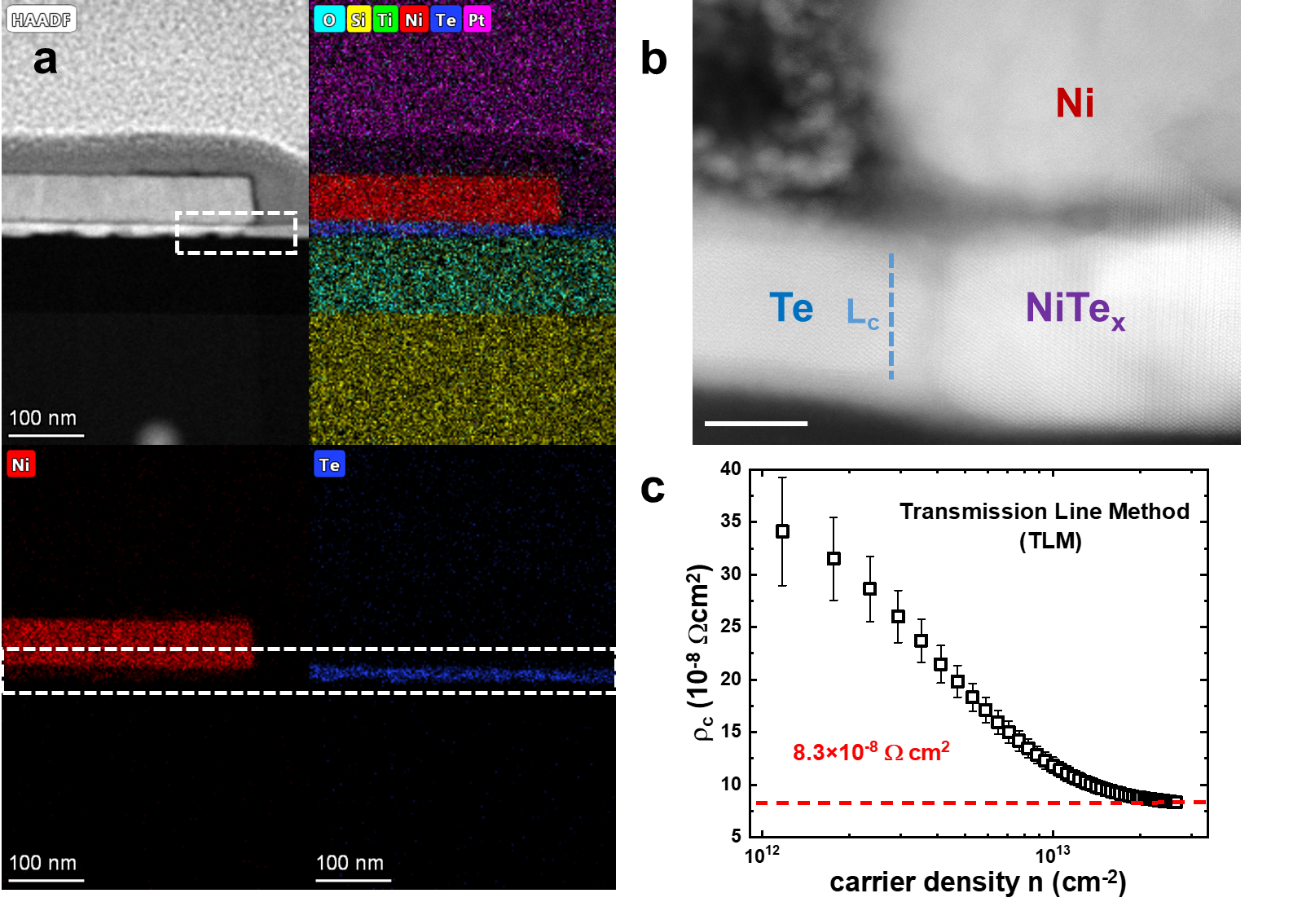


**Figure S1. Intimate NiTe_x_-Ni contacts. a**, HAADF-STEM images, and EDS elemental mapping of the 2D Te devices under Ni contact. The contrast different between Te under Ni and Te channel is highlighted. The diffusion of Ni is observed in EDS mapping. **b**, STEM image of the contact to channel interface. The interface is atomically sharp. **c**, Contact resistivity at different carrier densities of a 2D Te p-FET extracted using the transmission line method (TLM).


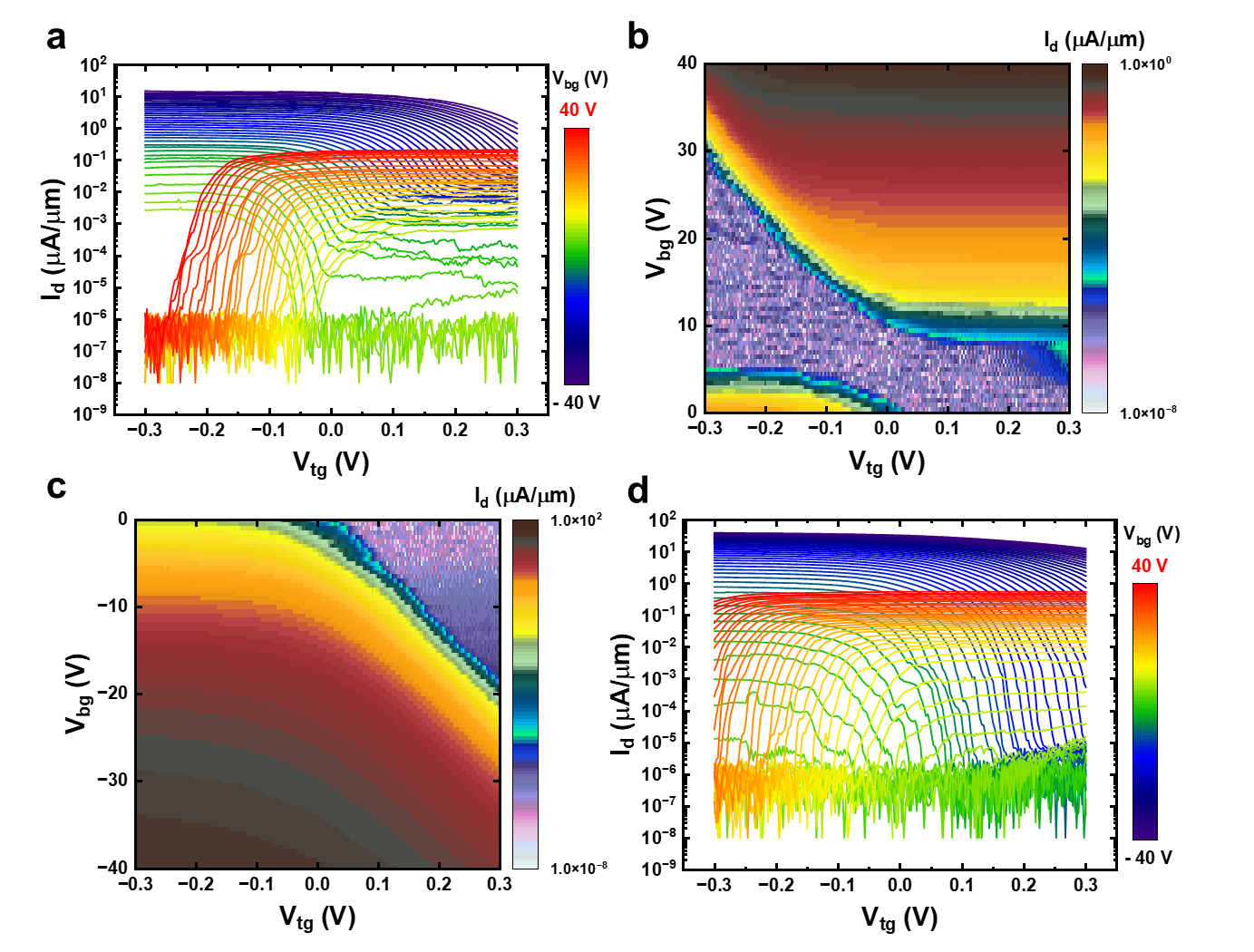


**Figure S2. Top-gate and back-gate dependence of the transfer characteristics. a**, Back gate voltage dependence of transfer characteristics of the same device in Figure 2a and 2b. **b**-**d**, Another similar 2D Te dual gate device with similar behavior, showing large top-gate tunability.


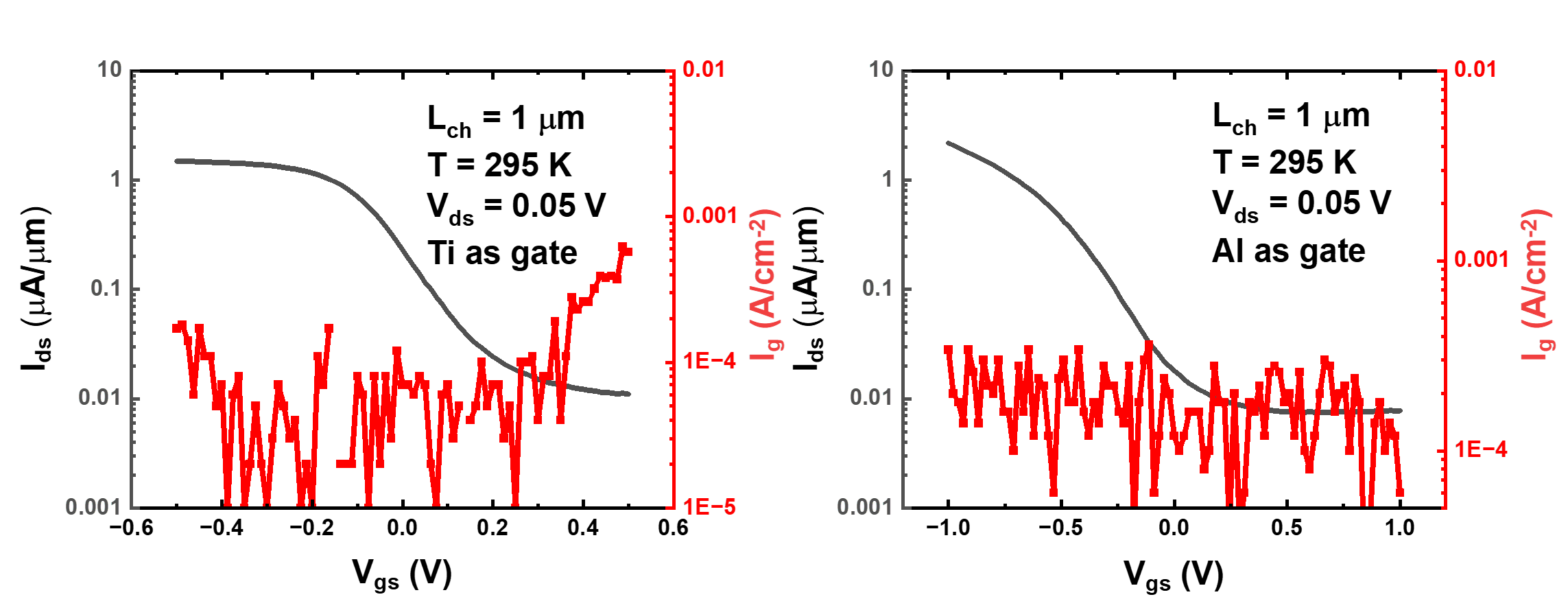


**Figure S3. Room temperature device behavior with Ti and Al as gate.** Transfer characteristics of Te-Ti Te-Al devices with gate leakage current normalized using the top gate area. Al shows better leakage current than Ti.


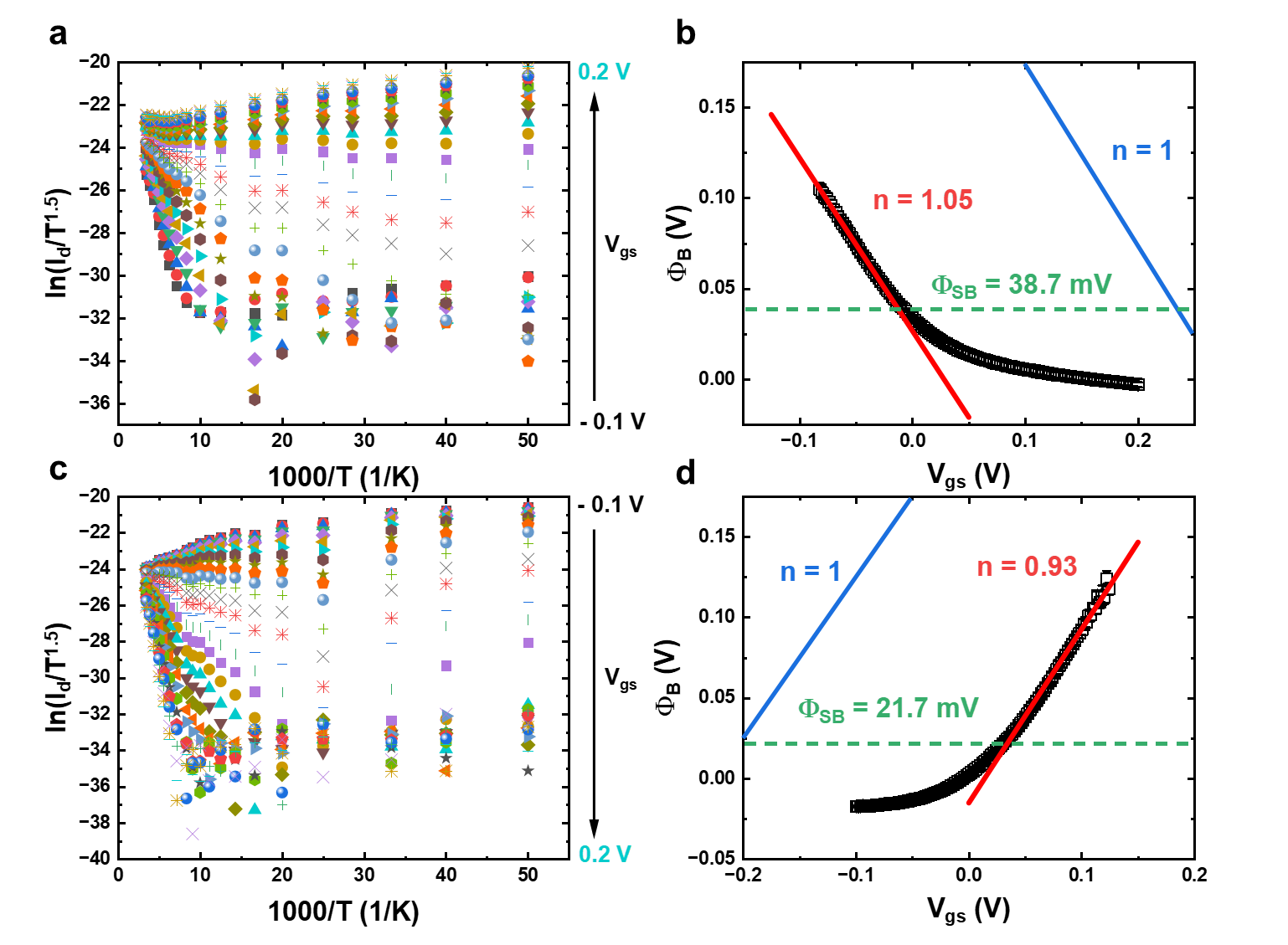


**Figure S4. Schottky barrier extraction for n-FET and p-FET. a**, Arrhenius plot at different gate voltages for an n-type 2D Te FET. **b**, Gate-voltage dependence of the barrier height extracted from the slope of the Arrhenius plot at high temperatures. The flat band condition is determined using linear fitting. A Schottky barrier of 38.7 mV is extracted. **c**, Arrhenius plot at different gate voltages for a p-type 2D Te FET. **d**, A Schottky barrier of 21.7 mV is extracted.


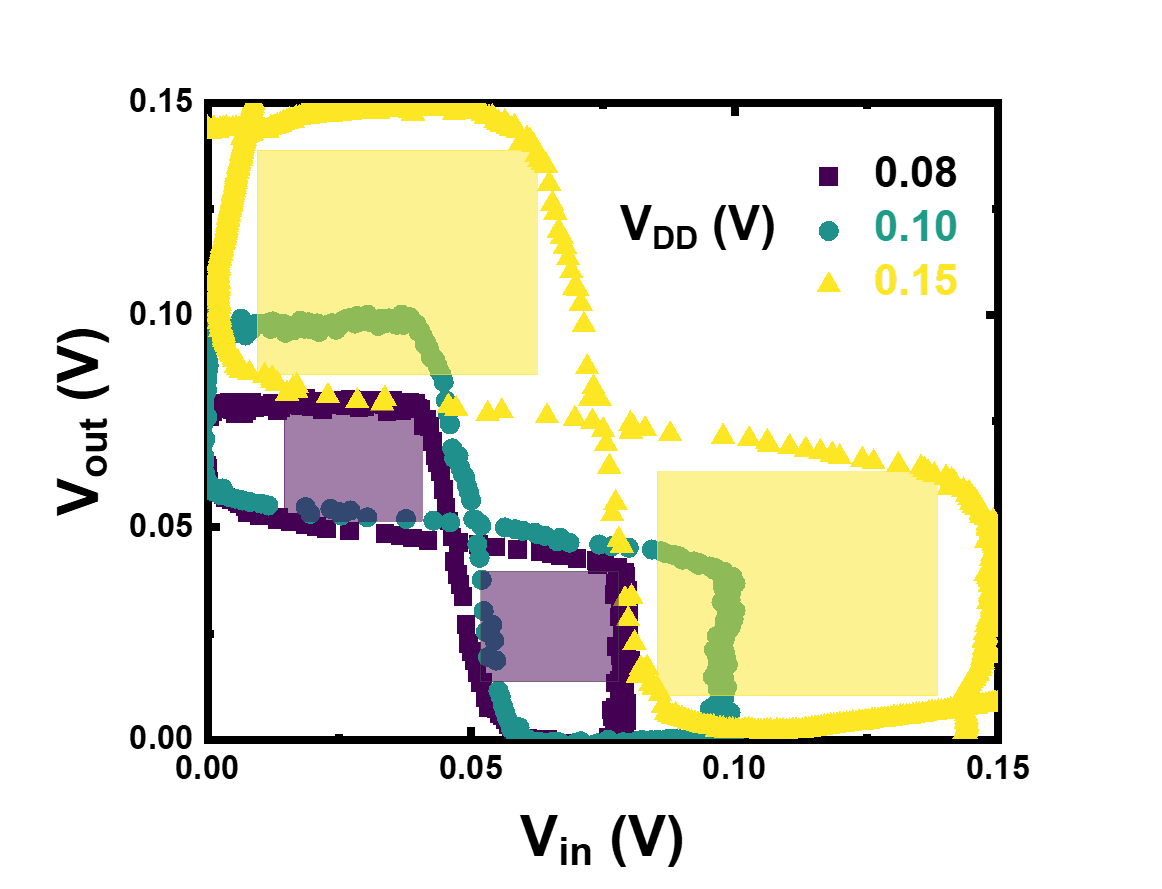


**Figure S5. Noise margin extraction of the CMOS inverter.** Butterfly curves under different supply voltages. The noise margin is extracted using the largest possible square method.
